# Intimate Partner Violence Among Brazilian Trans and Cisgender Women Living with HIV or at HIV Risk During COVID-19 Era: Another Epidemic?

**DOI:** 10.1089/trgh.2023.0057

**Published:** 2025-02-10

**Authors:** Ricardo de Mattos Russo Rafael, Emilia M. Jalil, Luciane de Souza Velasque, Ruth Khalili Friedman, Michelle Ramos, Cynthia B. Cunha, Eduardo Mesquita Peixoto, Lívia Machado de Mello Andrade, Davi Gomes Depret, Adriana Costa Gil, Dandara Costa Alcântara, Laylla Monteiro, Virginia Maria de Azevedo Oliveira Knupp, Valdiléa G. Veloso, Erin C. Wilson, Beatriz Grinsztejn

**Affiliations:** ^1^ Department of Public Health Nursing, College of Nursing, State University of Rio de Janeiro, Rio de Janeiro, Brazil.; ^2^ Evandro Chagas National Institute of Infectious Diseases, Oswaldo Cruz Foundation, Rio de Janeiro, Brazil.; ^3^ Department of Quantitative Methods, Federal University of Rio de Janeiro State, Rio de Janeiro, Brazil.; ^4^ College of Nursing, Federal University of Rio de Janeiro State, Rio de Janeiro, Brazil.; ^5^ Medical School, Federal University of Rio de Janeiro State, Rio de Janeiro, Brazil.; ^6^ College of Nursing, Federal University of the State of Rio de Janeiro, Rio de Janeiro, Brazil.; ^7^ Center for Public Health Research, San Francisco Department of Public Health, San Francisco, California, USA.; ^8^ Department of Epidemiology and Biostatistics, University of California, San Francisco, California, USA.

**Keywords:** cisgender women, COVID-19, HIV, intimate partner violence, trans women

## Abstract

**Purpose:**

Our study was conducted to estimate intimate partner violence (IPV) prevalence and associated factors among trans and cisgender women at risk of or living with HIV during the coronavirus disease (COVID-19) pandemic in Rio de Janeiro, Brazil.

**Methods:**

A cross-sectional study was conducted between May and August 2020 through telephone surveys with cisgender and trans women enrolled in two cohort studies in Rio de Janeiro. We assessed IPV employing the Revised Conflict Tactics Scale during the initial months of shelter-in-place ordinances. Regression models evaluated the factors associated with IPV for each population.

**Results:**

We surveyed 796 women, and 341 participants (47.78%) were eligible and included in the current analysis. All cisgender women and 41 (64.06%) trans women were living with HIV. Overall IPV prevalence was 27.86% (95% confidence interval [95% CI] 23.34–32.88). IPV was 63% higher among trans women than among cisgender women (prevalence ratio 1.63, 95% CI 1.14–2.34, *p*=0.008). Loneliness was significantly associated with IPV in both groups. Younger age and binge drinking were associated with IPV prevalence among trans women. For cisgender women, IPV was associated with withdrawal of cash transfer programs during the shelter-in-place.

**Conclusion:**

Trans women experienced significantly more IPV than cisgender women in the early phase of the COVID-19 epidemic. Plans to prevent and address violence against cisgender and trans women, especially those with heightened vulnerability that may be associated with living with HIV, are needed in public health planning for future pandemics.

## Introduction

Intimate partner violence (IPV) and HIV infection are public health problems with significant interactions. According to the World Health Organization (WHO), ∼20% of cisgender women worldwide have suffered physical or sexual violence at least once in their lives, especially in low- and middle-income countries.^1^ Studies with trans women around the globe estimate the median lifetime prevalence of IPV to be 37.5%.^2^ IPV may increase the risk of acquiring HIV, and women living with HIV infection may be at increased risk of experiencing IPV.^1^

IPV rates tend to increase during humanitarian crises, as was the case during the peak of the coronavirus disease (COVID-19) pandemic.^3^ In fact, the international community issued warnings about a potential IPV increase during the COVID-19 pandemic.^4–6^ However, IPV increases are not distributed equally across populations during a crisis. Research shows that IPV among cisgender women increases in the aftermath of disasters, often because of the underlying social vulnerability they face.^7^ Less is known about the IPV for trans women in emergencies, but given their high social vulnerability and significant baseline IPV experiences, instances of violence may get worse in a disaster situation.

Violence is a complex phenomenon, with cultural and historical ties and manifestations in each society.^8,9^ According to the Theory of Coercive Control, IPV is not simply a set of conflict events or physical aggression, but rather a phenomenon of control and oppression most often exerted by men over their intimate partners, with the goal of maintaining power and dominance over them.^10^ However, there are distinct risk factors that predict vulnerability to IPV across settings, such as age, gender (i.e., cisgender and trans women), health conditions (e.g., HIV infection), substance abuse, and unemployment.^11–13^

In the Brazilian context, the 2019 National Health Survey revealed that 7.60% of cisgender women reported having experienced violence at least once in their lives.^14^ While specific research on IPV estimates prevalence rates ranging from 20% to 50% for cisgender women,^15,16^ research with trans women in Brazil indicates even higher prevalence rates.^17–19^ Brazil holds the unfortunate distinction of being the country with the highest number of murders of trans women worldwide, leading this ranking for over a decade.^20^

Concerns about IPV were elevated due to the shelter-in-place strategy for COVID-19 that severely limited cisgender and trans women's ability to escape violent living situations. IPV increases during disasters have been attributed, in part, to the economic impact that can cause strain on relationships. However, more research is needed to determine factors associated with IPV for cisgender and trans women in the context of a global pandemic, hence future plans to prevent exacerbation of existing vulnerabilities can be developed.^21^

Despite warnings, little monitoring was conducted on IPV prevalence during the pandemic, and what was reported focused mainly on cisgender women attending emergency services and law enforcement reports.^22,23^ Dedicated research is needed to determine the scope of IPV exposure for trans and cisgender women during the COVID-19 epidemic, and to identify factors associated with elevated IPV in this type of disaster context. For this study, we aimed to estimate IPV prevalence at the community level, and to identify its associated factors among trans and cisgender women at risk of or living with HIV during the COVID-19 pandemic in Rio de Janeiro, Brazil.

## Materials and Methods

### Design and sample

This is a cross-sectional study enrolling trans and cisgender women followed in two cohort studies conducted at the Evandro Chagas National Institute of Infectious Diseases (INI), Oswaldo Cruz Foundation (FIOCRUZ). The Women's HIV Cohort at INI is an observational study of cisgender women living with HIV, established in 1996.^19^ The *Transcendendo* Cohort is prospective, open cohort to longitudinally evaluate health outcomes among trans women living with HIV or at HIV risk since 2015.^18^ Any participant from both cohorts could be invited to answer the survey.

The inclusion criteria for the current analysis were as follows: (1) currently living in Rio de Janeiro or the metropolitan area, (2) previous consent to contact, (3) phone number availability, (4) having an intimate partner in the period of social distance, and (5) answer questions about the outcome (IPV). This study was approved by the INI Institutional Review Board. Informed consent was obtained from all participants during the development of the cohorts. Contacted telephone numbers were already on record in relation to topics regularly monitored in previous studies.

### Data collection

Trained interviewers contacted potential participants by phone at least three times to invite them for participation in a remote survey between May 19 and August 21, 2020. Phone attempts occurred on different days and times, including weekends. If attempts were not successful, participants were marked as unreachable. For those who refused to participate, there was no further contact.

To ensure participant safety, interviewers told potential participants that this was a survey of IPV and asked if they could safely answer the survey questions during the phone interview (e.g., they were in a private space in which their partners could not hear their responses). If the interviewers determine that potential participants were not in a safe space to be interviewed, they were offered the option to postpone the survey or schedule an in-person visit to assess the situation.

### Measurements

The remote survey was hosted on the Shiny application, and contained questions on demographics, substance use, sexual behavior, and social distancing. Demographics measures included age, number of people living in the house, family income (R$1=U$5.56 conversion rate), impact of the COVID-19 pandemic on income (difference between income before and during the pandemic period), participation in the cash transfer program (CTP) called *Bolsa Familia* before and after the epidemic began, binge drinking (6+ alcohol doses in one occasion), and drug use.

Race/color, gender identity, and HIV status were retrieved from cohort databases. We used the Brazilian Loneliness Scale, a validated and crossculturally adapted version of the Revised UCLA Loneliness Scale,^24–26^ to evaluate loneliness. Minimal, mild, and moderate/severe ranges were, respectively, up to 22, 23–35, and 36–47 points.^27^

We assessed IPV using the Revised Conflict Tactic's Scale (CTS2), which was validated and crossculturally adapted for use in Brazil.^28,29^ CTS2 measures 39 behaviors divided into three categories: negotiation, violence, and injury. Violence was measured with three subscales (violence types): psychological, physical, and sexual violence, and two severity levels (“severe” and “minor”). CTS2 was considered as severe IPV threats, use of force, or use of weapons.

We adjusted the time frames of the CTS2 survey to align with the onset of the shelter-in-place recommendation. Therefore, the items related to violence referred to events up until March 16, 2020. The study outcome was defined as experiencing IPV, which was operationalized as a participant answering “yes” to any one or more items on the CTS2. We evaluated psychological, physical, and sexual violence in addition to overall IPV. Severe IPV was defined for each violence type (psychological, physical, and sexual violence) as the presence of at least one item from the severity subscale.

### Statistical analysis

Descriptive statistics were calculated using the mean (standard deviation [SD]) or median (interquartile range [IQR]), and absolute and relative counts, as appropriate. We estimated IPV prevalence and its 95% confidence intervals (95% CIs). Bivariate analyses were conducted using Fisher's exact test for categorical variables and two-sided Student's *t*-test and Wilcoxon Rank Sum for numerical variables according to the Shapiro–Wilk normality test.

We analyzed a different regression model for each population (i.e., cisgender women and trans women). Multivariable models included variables with a *p*-value up to 0.30. The final model was comprised of variables with a *p*-value <0.05 after a stepwise backward Poisson regression analysis with robust variance to detect factors associated with IPV. All analyses were performed on Stata SE version 15.^30^

## Results

**FIG. 1.**
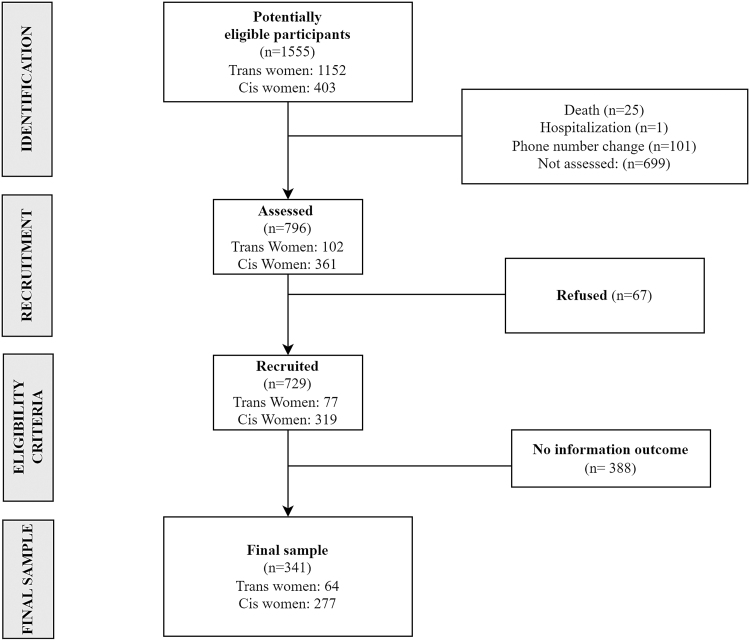
Study flowchart. Rio de Janeiro, Brazil, 2020.

Figure 1 shows the study flowchart. Using the cohort databases, we identified 1555 potentially eligible participants (1152 cisgender women [74.08%] and 403 [25.92%] trans women) and contacted 796 participants (601 cisgender women [52.17%], 195 trans women [48.39%], 51.19% response rate). It is worth noting that there were 632 women who were not assessed because the team did not get a response from them despite multiple attempts to contact them at different times and days of the week.

Among contacted individuals, 729 (46.88%) were interviewed: 559 (76.68%) cisgender women and 170 (23.32%) trans women. For the present analysis, 341 (42.84%) were eligible: 277 (81.23%) cisgender women and 64 (18.77%) trans women. Main reasons for ineligibility were no intimate partner during assessed period (333/388 [85.82%]) and no information on the violence outcome (55/388 [14.18%]) due to refusal to answer questions related to IPV.

Table 1 describes the sample characteristics. Overall, participants had a mean age of 44.10 (SD 10.20) years, most participants were Black/Brown (66.67%), and the median number of people living in the house during social distancing was 2 (IQR 1–3). Median family income per month was U$ 341.73 (IQR U$ 197.84–539.57). Few (12.61%) participants reported an impact of COVID-19 on their monthly income during the social distancing period, and 34.32% participated in the CTP at some time in their lives.

**Table 1.** Characteristics of Study Population According to Gender Identity, Rio de Janeiro, Brazil, 2020

**Variables**	**Overall (*n*=341)**	**Cisgender women (*n*=277)**	**Trans women (*n*=64)**	***p***
Age,^a^ mean (SD)	44.10 (10.20)	46.19 (8.99)	35.05 (10.24)	**<0.001** ^*^
Race/color				
White	113/339 (33.33)	99 (35.74)	14/62 (22.58)	0.053
Black/brown	226/339 (66.67)	178 (64.26)	48/62 (77.42)	
Number of people living in the house,^b^ median (IQR)	2 (1–3)	2 (1–3)	1 (0–2)	**<0.001** ^**^
Family income, median (IQR)	341.73 (197.84–539.57)	359.71 (215.83–539–56)	197.84 (125.90–295.50)	**<0.001** ^**^
COVID-19 impact on income^c^				
No	298 (87.39)	244 (88.09)	54 (84.38)	0.408
Yes	43 (12.61)	33 (11.91)	10 (15.63)	
Participation on CTP^d^				
Never	222/338 (65.68)	196/275 (71.27)	26/63 (41.27)	**<0.001**
Withdrawn during social distancing	8/338 (2.37)	7/275 (2.55)	1/63 (1.59)	
Maintained during social distancing	37/338 (10.95)	28/275 (10.18)	9/63 (14.29)	
Begun during social distancing	71/338 (21.01)	44/275 (16.00)	27/63 (42.86)	
Loneliness				
Minimum	256/336 (76.19)	218/273 (79.85)	38/63 (60.32)	**0.003**
Mild	45/336 (13.39)	33/273 (12.09)	12/63 (19.05)	
Moderate/severe	35/336 (10.42)	22/273 (8.06)	13/63 (20.63)	
Binge drinking^e^				
No	276/340 (81.18)	237 (85.56)	39/63 (61.90)	**<0.001**
Yes	64/340 (18.82)	40 (14.44)	24/63 (38.10)	
Any drug use				
No	292/335 (87.16)	250/272 (91.91)	42/63 (66.67)	**<0.001**
Yes	43/335 (12.84)	22/272 (8.09)	21/63 (33.33)	
HIV status				
HIV risk	23 (6.74)	—	23 (35.94)	**<0.001**
HIV positive	318 (93.26)	277 (100.00)	41 (64.06)	

The bold values denote *p*-values <0.05.

^*^ *t*-Student 2 sides.

^**^ Wilcoxon and Mann–Whitney.

^a^ Continuous variable.

^b^ Number of people living with the participant during COVID-19 pandemic.

^c^ The variable was generated from the difference between current income and income before the pandemic.

^d^ The variable represents the extreme social vulnerability when a subject receipt of donations from the minimum set of foods to feed a family.

^e^ 6+ alcohol doses in one occasion.

CTP, cash transfer program; IQR, interquartile range; SD, standard deviation.

About one-quarter (23.81%) of the participants reported feeling lonely, while 10.42% (35/336) had moderate-to-severe feelings of loneliness during the pandemic. HIV infection was present in 93.26; (318) of the participants, with all cisgender women being seropositive due to eligibility criteria of the parent study from which participants were recruited, and 41 trans women (64.06%). It was observed that cisgender and trans women had significant differences in all demographic characteristics, except for race/color and income impact, which reinforces the need for distinct models in comparing factors associated with IPV.

Overall, IPV prevalence was 27.86% (95% CI 23.34–32.88) and 63% higher among trans women than among cisgender women (prevalence ratio [PR] 1.63, 95% CI 1.14–2.34, *p*=0.008). Trans women had ∼3 times higher prevalence of severe IPV compared with cisgender women (PR 2.96, 95% CI 1.54–5.68, *p*=0.001). Disaggregating the IPV components, psychological, physical, and sexual violence prevalence was higher among trans women than for cisgender women (Table 2). Both severe psychological and physical IPV were significantly higher in trans women than in cisgender women (respectively, PR 2.97, 95% CI 1.45–6.12, *p*=0.003, and PR 5.41, 95% CI 1.49–19.62, *p*=0.010).

**Table 2.** Intimate Partner Violence Prevalence Overall and According to Type, Severity Level, and Gender Identity During the COVID-19 Pandemic in Rio de Janeiro, Brazil, 2020

**Variables**	**Overall (*n*=341), *n* (%)**	**Cisgender women (*n*=277), *n* (%)**	**Trans women (*n*=64), *n* (%)**	***p***
IPV	95 (27.86)	69 (24.91)	26 (40.63)	0.014
Minor	91 (26.69)	66 (23.83)	25 (39.06)	0.018
Severe	32 (9.38)	19 (6.86)	13 (20.31)	0.002
Psychological violence	91 (26.69)	67 (24.19)	24 (37.50)	0.041
Minor	87 (25.51)	64 (23.10)	23 (35.94)	0.039
Severe	27 (7.92)	16 (5.78)	11 (17.19)	0.008
Physical violence	18 (5.28)	8 (2.89)	10 (15.63)	<0.001
Minor	16 (4.69)	7 (2.53)	9 (14.06)	0.001
Severe	9 (2.64)	4 (1.44)	5 (7.81)	0.014
Sexual violence	17 (4.99)	7 (2.53)	10 (15.63)	<0.001
Minor	16 (4.69)	6 (2.17)	10 (15.63)	<0.001
Severe	4 (1.17)	2 (0.72)	2 (3.13)	0.161

IPV, intimate partner violence.

**FIG. 2.**
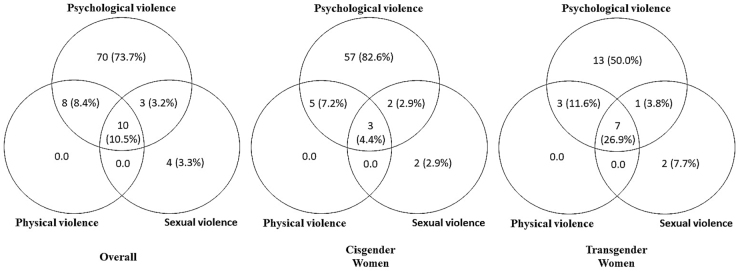
Interconnections between Intimate Partner Violence according to types and gender identity during the COVID-19 pandemic in Rio de Janeiro, Brazil, 2020.

Figure 2 shows that 10.5% of women who experienced IPV during the social distancing period reported all the three violence types simultaneously, with significantly more trans women reporting all three types of violence experienced during that period (26.9% vs. 4.4%; *p*=0.007). Psychological violence was the most common IPV type and overlapped with most of other violence categories.

Multivariable analysis identified a significant association between loneliness and IPV in both groups (Table 3). Cisgender women with mild and moderate/severe loneliness had IPV prevalence of, respectively, 2.50 (95% CI 1.60–3.93, *p*<0.001) and 2.76 (95% CI 1.70–4.49, *p*<0.001) higher than those with minimal loneliness. Among trans women, we observed similar association between mild (PR 2.35, 95% CI 1.64–3.38, *p*≤0.001) and moderate/severe loneliness (PR 2.24, 95% CI 1.45–3.36, *p*<0.001) and IPV. Cisgender women withdrawn from CTP during the social distancing period had 3.56 times the IPV prevalence (95% CI 2.17–5.87, *p*<0.001) as compared with those who never received it.

**Table 3.** Crude and Adjusted Intimate Partner Violence Prevalence Ratios By Gender Identity During the COVID-19 Pandemic in Rio de Janeiro, Brazil, 2020

	**Cisgender women**				**Trans women**			
	**cPR (95% CI)**	***p***	**aPR (95% CI)**	***p***	**cPR (95% CI)**	***p***	**aPR (95% CI)**	***p***
Age^a^	0.99 (0.97–1.01)	0.556			0.96 (0.92–0.99)	**0.040**	0.98 (0.97–0.99)	**0.047**
Race/color								
White	Reference	—			Reference	—		
Black/Brown	0.98 (0.64–1.50)	0.922			1.53 (0.62–3.75)	0.351		
Number of people living in the house^b^	1.13 (1.00–1.28)	0.043			1.13 (0.91–1.40)	0.277		
Familiar income	1.00 (0.99–1.00)	0.745			1.00 (0.99–1.00)	0.098		
Impact on income^c^								
No	Reference	—			Reference	—		
Yes	1.10 (0.61–2.02)	0.735			0.70 (0.26–1.92)	0.494		
Participation on cash transfer program^d^								
Never	Reference	—	Reference	—	Reference	—		
Withdrawn during social distancing	4.20 (2.78–6.33)	**<0.001**	3.56 (2.17–5.87)	**<0.001**	—	—		
Maintained during social distancing	1.05 (0.49–2.25)	0.900	0.92 (0.42–2.01)	0.835	0.78 (0.28–2.22)	0.652		
Begun during social distancing	1.78 (1.10–2.88)	**0.018**	1.53 (0.96–2.45)	0.072	1.05 (0.56–1.95)	0.876		
Loneliness								
Minimum	Reference	—	Reference	—	Reference	—	Reference	—
Mild	2.80 (1.82–4.33)	**<0.001**	2.50 (1.60–3.93)	**<0.001**	1.46 (0.71–3.01)	0.304	2.35 (1.64–3.38)	**<0.001**
Moderate/severe	2.97 (1.85–4.77)	**<0.011**	2.76 (1.70–4.49)	**<0.001**	1.57 (0.80–3.08)	0.187	2.24 (1.45–3.36)	**<0.001**
Binge drinking^e^								
No	Reference	—			Reference	—	Reference	—
Yes	1.37 (0.83–2.27)	0.214			2.60 (1.41–4.78)	**0.002**	1.45 (1.04–2.03)	**0.030**
Any drug use								
No	Reference	—			Reference	—		
Yes	1.54 (0.85–2.80)	0.156			2.00 (1.13–3.53)	**0.017**		
HIV status								
HIV risk	—	—	—	—	Reference	—		
HIV positive	—	—	—	—	1.06 (0.56–1.99)	0.857		

The bold values denote *p*-values <0.05.

^a^ Continuous variable.

^b^ Number of people living with the participant during COVID-19 pandemic.

^c^ The variable was generated from the difference between current income and income before the pandemic.

^d^ The variable represents the extreme social vulnerability when a subject receipt of donations from the minimum set of foods to feed a family.

^e^ 6+ alcohol doses in one occasion.

aPR, adjusted prevalence ratio; CI, confidence interval; cPR, crude prevalence ratio.

IPV and participation in CTP were not associated for cisgender women who maintained their participation in CTP during the pandemic. Among trans women, both age and binge drinking were associated with IPV prevalence. Younger trans women had significantly higher IPV prevalence (*p*=0.047). IPV prevalence was 30% higher in trans women who reported binge drinking during the social distancing period compared with those who did not (*p*=0.030).

## Discussion

We found that about one-third of all women experienced IPV during the COVID-19 pandemic, and minor and severe IPV prevalence among trans women was significantly higher than that among cisgender women. The findings of our study align with global data on domestic violence, suggesting that ∼30% of women have experienced physical and sexual violence in their lifetime, as reported by the WHO. This prevalence ranges from 23.2% in high-income countries to 37.0% in the Eastern Mediterranean and Southeast Asian regions.^31,32^ When comparing these results with data from Brazil, the findings of our study become even more concerning.^14,23,33–38^

Trans-specific studies also shed light on the dramatic reality experienced by trans women during the COVID-19 pandemic in Brazil. The *Transcendendo* Cohort, conducted in Brazil between 2015 and 2017, found high rates of lifetime physical and sexual violence at 54.0% and 46.3%, respectively.^18^ Data from Brazil are consistent with studies internationally that found disproportionately higher IPV rates among trans individuals compared with cisgender individuals.^2,38^

Results of our study demonstrate the extreme vulnerability to violence that trans women in Brazil faced during the peak months of the COVID-19 pandemic, as the prevalence of IPV we found during first months of shelter-in-place ordinances approached or exceeded the prevalences observed in Brazilian and global investigations over a lifetime.

We observed a significant correlation between feelings of loneliness and domestic violence in both groups of women surveyed for our study. Loneliness has been linked to detrimental health outcomes, such as depression and suicidal tendencies.^39,40^ It is important to note that loneliness is not a novel phenomenon nor exclusively tied to the COVID-19 pandemic. It is not surprising to note that certain studies have referred to it as a “behavioral epidemic,” highlighting the association between loneliness and social isolation in periods predating the pandemic, particularly in Europe, the United States, and China.^41^

However, it is important to reflect on how the shelter-in-place restrictions imposed to contain the pandemic may have intensified the effects of these negative feelings. The disruption in participation in community, work, or study activities may have contributed to feelings of loneliness.^42,43^ These feelings of loneliness may have pushed women into relationship situations they would not have otherwise tolerated; in addition or conversely, isolation may have been used against women as a tool of control and to facilitate IPV.

The pandemic, paired with shelter-in-place requirements, may have also increased tensions and stress, limited access to help for victims, intensified the exercise of control and power by perpetrators, and reduced surveillance and protection by the government and the community. These combined factors may have significantly contributed to the increase in abuse and violence against women,^44–47^ including trans women who already experienced violence and exclusion from protections in Brazilian public policies.

These circumstances further amplify the challenges faced by victims and the urgency of comprehensive and inclusive efforts to combat gender-based violence. However, while this study does not seek to establish causal relationships, it is important to highlight that the feeling of loneliness, especially due to its strong association with violence, should be considered as a factor to be observed when identifying and suspecting episodes of violence against women. Being attentive to this aspect is crucial for a more comprehensive and effective approach to preventing and combating gender-based violence.

Excessive alcohol consumption (binge drinking) and the interruption of income through CTP were identified as factors that were also associated with IPV. Due to the temporal nature of the data, these relationships are hard to disentangle, but one hypothesis is that binge drinking was associated with violence as a coping mechanism. That is, women who experienced violence may be drinking excessively to cope with the distress caused by IPV.

Women not on the CTP may have been reliant on their partners for income, which could be another indicator of control and violence that cisgender women were facing. Both of these factors point to a need to better understand the vulnerabilities faced by many women during the pandemic so policies to protect the general public are implemented in ways that also serve the most vulnerable in our communities, including women at risk of violence.

### Limitations

Our study has some limitations. First, we used convenience sampling, and thus results may not be generalizable to the whole population of trans and cisgender women in Brazil. An important bias in the sample was that all cisgender women were living with HIV, which limits inference between HIV and violence in this population. Thus, the current analysis did not identify an association between IPV and HIV infection.

Nevertheless, most of our samples were living with HIV, and we could only evaluate HIV infection as a covariable among trans women, as all cisgender women were living with HIV. As such, we may have been underpowered to detect a potential association as several studies have described the synergistic associations between HIV infection and IPV.^1^ Moreover, severe IPV types have been associated with avoidance of health services. Since all women enrolled in the study were health care users, our sample may be biased to not adequately capture women experiencing severe violence.

Also, we may not infer any causal associations due to the cross-sectional design. The high IPV estimates among trans women may have overestimated overall IPV rates. HIV infection may also have underestimated IPV rates among cisgender women.

In contrast, IPV prevalence may have been underestimated as we restricted interview eligibility to women who were in a private setting due to safety concerns. There is always concern on IPV disclosure, and the remote survey may increase negative answers. Finally, it is important to note that the shelter-in-place period may have impacted the study participants differently. It is possible that the women interviewed at the outset of the investigation had less exposure compared with those interviewed later in August 2020.

Although this study deals with data from the beginning of the COVID-19 pandemic, it is important to highlight that these were the months of greatest shelter-in-place in Brazil and, consequently, a moment of greater risk of violence against women. Thus, despite the limitations, this study brings important contributions.

## Conclusion

Although shelter-in-place measures are needed for the safety of society overall, they may have acute negative impacts on already vulnerable populations through increased interactions among couples in a restricted setting, negative financial impact, and increased fear and anxiety related to COVID-19. Heightened stressors as a result of the pandemic may have contributed to the high IPV rates observed in our study. Current findings reinforce concerns about IPV during the COVID-19 pandemic and the consequences for the coming years, especially among vulnerable groups such as women in the context of HIV.

## Abbreviations Used

aPR: adjusted prevalence ratio
cPR: crude prevalence ratio
CI: confidence interval
COVID-19: coronavirus disease
CTP: cash transfer program
CTS2: Revised Conflict Tactic's Scale
INI: Evandro Chagas National Institute of Infectious Diseases
IPV: intimate partner violence
IQR: interquartile range
PR: prevalence ratio
SD: standard deviation
WHO: World Health Organization
